# Lock Jaw and Tetanus

**Published:** 1809-10

**Authors:** John Howship

**Affiliations:** Surgeon


LOCK JAW *and* TETANUS; *by* JOHN HOWSHIP, Surgeon.

*A Case of Tetanus, in which there was the most favourable Opportunity for ascertaining the good Effect of Pressure, either in Cases of Tetanus, or of Epilepsy, where the Disease is consequent to remote and local Irritation in the System.*

( In Continuation. )

ON the 23d of October 1805, his Majesty's ship Neptune, while cruizing off Cadiz, lost her foretop-mast in a squall. To get up another, it was necessary to bring the ship too, and the tackles not being perfectly cleared, in letting go the anchor, one of the men had his thumb very severely jammed, being drawn into the block at the cat-head.

James Warring, 20 years of age, a very robust strong man, had received this contusion on the thumb of his right hand. There were two lacerated wounds, one small in comparison with the other, the deepest having exposed the bone. The organization of the parts had suffered so severely from the contusion, that on the evening of the 17th day afterwards, when he was brought on shore to the Naval Hospital, Gibraltar, there did not appear to be any the slightest disposition to suppuration in the wounds.

On the 15th day he had been seized with pains, which his surgeon stated were confined at that time to his breast; so free was he supposed to be from spasmodic complaint, that his disorder was considered to be pulmonic inflammation, and therefore when he first spoke of his pain and difficulty of breathing, ℥xij. of blood were taken from him. A very short time, however, threw more light upon the true nature of the case, and in the two days that passed between the time of his first complaining and that of his being brought on shore, he had taken ℈ij. of solid opium; besides which, he had had strong solutions of opium applied as embrocations about the affected parts.

On the evening of the 17th, when first brought into the hospital, his jaws were closely knit together, although more rigidly fixed while the fits of increased violence of spasm were upon him. These exacerbations of pain and spasm were not of long continuance, and returned after the short interval of half a minute only. The characters of the complaint in this particular instance, differed somewhat from those frequently observed in other cases, in relation to the course and situation of the spasmodic twitches.

The evident exciting cause of the disease being in this instance so distant from the centre of the nervous system, little little doubt was entertained of the possibility of instituting such a degree of pressure as would effectually prevent the passing up of the irritating impression from the wound; at any rate it was determined to make the experiment, and a strong screw tourniquet was therefore applied in the usual manner, rather high up on the arm.

The instrument was screwed pretty tight, so that the patient made complaint on account of its pressure, but the convulsive twitches still continued in violence and frequency unaltered, so that every one of the medical gentlemen present agreed, that, as yet, the spasms were not in the least relieved.

The manner of attack was very uniform, and nearly this, with horrible pain; the angles of the lips were drawn back, the jaws held most closely together, and the body and lower extremities so contracted as to be raised up completely from the bed into the form of an arch, the points of support being the occiput and the heels. The whole series of muscles, posterior to the spine and limbs, being in this case affected by the same spasm.

The approach of this most terrible pain is first perceived in the generality of cases of tetanus, by the respiration becoming noisy and laborious, as well as by the moans of the unhappy sufferer.

The tourniquet seemed to have given no relief whatever, but as the last degree of pressure had not yet been produced, the screw was now turned tighter, and still tighter, till it was not possible to move it any farther.

This last operation created very great pain, to so intense a degree, that the poor man cried with agony, and begged for God's sake, that the band might be slackened. While the whole of the soft parts of the arm within the circle, were to the greatest possible degree compressed, he at first suffered three attacks of spasm with the interval of 30 seconds; these were more severe, as to pain, violence, and extent, than any he had before experienced. A much less severe spasm then followed: he lay at rest for two minutes, when a slight twitch was again felt. In this way he continued to feel them for about ten minutes; all the spasms, latterly, being manifestly less violent than they had been prior to the compression of the limb.

The man was himself questioned, and said, yes, he was relieved, and did not feel so much severity in the spasms, as he did before the instrument was last tightened.

In this manner was the experiment made, and thus it was ascertained, how far pressure or compression is capable of procuring relief in certain states of morbid irritation in nerves.

Pressure, in this case, had certainly been productive of relief, in having considerably diminished the severity of pain, and blunted the sharp edge of suffering; but it is at least equally clear, that the limb would have been eventually and very quickly destroyed, had this degree of pressure been continued.

From the satisfactory nature of the experiment, both in the effects on the body, and those upon the limb, it was considered perfectly unnecessary to lengthen out the continuance of this destructive pressure beyond ten minutes, when the screw was turned back, and the band slackened; it is curious, that the moment the effect of the instrument was diminished by the back turn of the screw, the same moment a spasm came on, in precisely the same degree of violence as before the application of the tourniquet, and very much more severe than those felt latterly, during the establishment of the compression.

The tourniquet was now removed from off the arm, and by the time this was accomplished, it was perceived that every character, both as to period and expression of the spasm, had resumed exactly its former appearances, the exacerbation of convulsion returning as before with only half minute intervals.

Upon the same day (the 17th) mercury was ordered, and the ungt. hydr. was rubbed in on various parts of the body; it was exhibited internally also; the warm bath was at the same time directed, and the immersion to be repeated every second hour.

Between ten and eleven the same night, after a period of apparent ease, this man was observed to shake and tremble; this increased upon him till the convulsive tremour was so severe as to be compared to a violent attack of ague; during this time he was insensible, and upon the subsiding of the tremour the vital actions seemed to be perfectly exhausted; the skin cold and relaxed, the heat not returning, he soon after expired.

In the first period of the disease, while the patient was on shipboard, the cold affusion was tried repeatedly, and his own surgeon, who stated these particulars when the man was brought to the hospital, was convinced, with benefit and relief to the patient; why the same plan of treatment was not followed up is hard to say; perhaps the senior medical officer might have had a natural antipathy to cold water.

( To be continued. )

4, *Mill-street, Hanover-square,*
*Sept.* 16, 1809.

